# Editorial: Nutritional Assessment Tools for Identification and Monitoring of Malnutrition in Patients With Chronic Disease

**DOI:** 10.3389/fnut.2022.870514

**Published:** 2022-03-22

**Authors:** Eloisa Colin-Ramírez, Lilia Castillo-Martínez

**Affiliations:** ^1^Escuela de Ciencias del Deporte/Centro de Investigación en Ciencias de la Salud (CICSA), Facultad de Ciencias de la Salud, Universidad Anáhuac México Campus Norte, Huixquilucan, Mexico; ^2^Servicio de Nutriología Clínica, Instituto Nacional de Ciencias Médicas y Nutrición Salvador Zubirán, Facultad de Medicina, Universidad Nacional Autónoma de México, Mexico City, Mexico

**Keywords:** sarcopenia, muscle strength, nutrition status assessment, malnutrition, chronic diseases

Chronic disease-related malnutrition (DRM) is a highly prevalent condition that is associated with prolonged hospital stays, higher morbidity and mortality, and increased economic burden. Its prevalence has been reported to be between 20 and 50% depending on the patient population and the criteria used for its diagnosis (1). Due to its clinical and economic consequences, nutritional screening of patients with chronic diseases should be conducted for the early detection and management of malnutrition. However, nutritional evaluation is not a routine practice in the clinical setting, and in the absence of standardized procedures, several operative diagnostic definitions for DRM have been proposed. The European Society for Clinical Nutrition and Metabolism, in its most recent guidelines on definitions and terminology of clinical nutrition, endorsed the following definition of malnutrition “a state resulting from lack of intake or uptake of nutrition that leads to altered body composition (decreased fat-free mass) and body cell mass leading to diminished physical and mental function and impaired clinical outcome from disease” (2). In addition, this organization recognized the existence of a specific type of DRM with inflammation, which is characterized by a catabolic condition with an inflammatory response, including anorexia and tissue breakdown elicited by an underlying disease (2). This is the type of malnutrition commonly seen in heart failure, chronic kidney disease, liver disease, arthritis, and cancer. Tools such as biomarkers, e.g., serum concentrations of visceral proteins, may be not valid in the context of DRM with inflammation and should not be used as indicators of a patient's nutritional status. Fluid retention is a factor related to DRM with inflammation that may be hindering valid assessment of changes in body cell mass and nutritional status, as documented in heart failure and chronic kidney disease (3, 4). Thus, identifying additional nutritional assessment tools for the proper identification of patients with nutritional abnormalities is urgently needed.

Sarcopenia is recognized as a nutrition-related condition that may be related to the aging process (primary sarcopenia); however, it may also result from pathogenic mechanisms (secondary sarcopenia) that are disease-related, activity-related, or nutrition-related (2). This condition is characterized by a progressive and generalized skeletal muscle disorder that is associated with increased risk of adverse outcomes including falls, fractures, physical disability, and mortality (5).

This Research Topic addressed the current and novel nutritional assessment tools for identification and monitoring of malnutrition in patients with chronic disease. A total of 12 articles were published in this Research Topic covering different aspects of the above-mentioned topic. Sarcopenia was the topic of greatest interest; 5 out of 12 papers addressed this topic; including a systematic review aimed to explore the association between tongue strength and sarcopenia. This work showed that tongue strength is correlated with the subcomponents of sarcopenia, suggesting that sarcopenia is a systemic disease that may affect the skeletal muscles of the whole body, and that tongue pressure may be an indicator of subclinical dysphagia (Chen et al.). Also, data from Zhang, Zhang et al. highlighted the impact of sarcopenia and its muscle function and muscle composition components on clinical outcomes in an Asian population, while Mao et al. developed and evaluated the prognostic value of a novel index based on a combination of albumin-globulin score and sarcopenia in patients with renal cell carcinoma, showing a good performance of this index which reflects patient's nutritional and inflammatory status. Do et al. showed that phase angle assessed by the bioimpedance analysis technique is independently associated with muscle mass, strength, and sarcopenia in patients undergoing peritoneal dialysis, suggesting that phase angle can be used as a simple predictor of risk for sarcopenia in this patient population. Finally, Tejavath et al. reported results of a randomized clinical trial that demonstrated that long-term supplementation with branched-chain amino acids improved sarcopenia parameters and prognostic markers in elderly patients with advanced liver cirrhosis.

Four papers in this Research Topic provided evidence on the high prevalence of malnutrition and its prognostic value in diverse clinical conditions. Zhang, Qian et al. exhibited a high prevalence of malnutrition in elderly patients with cancer using three different scoring systems, ranging between 11.7 and 58.7%. Malnutrition was prevalent even in those who were overweight or obese as assessed by body mass index (BMI). Notably, malnutrition was associated with all-cause mortality regardless of the malnutrition index used, tumor types, and other risk factors, while deterioration of nutritional status was associated with deterioration in quality of life and immunotherapeutic response. Ding et al. also reported a high prevalence of patients at risk of malnutrition (77.76%) by using the NRS2002 screening tool and with mild/moderate malnutrition (10.09%) according to the Patient-Generated Subjective Global Assessment (PG-SGA) instrument among patients newly diagnosed with gastrointestinal stromal tumors. In patients undergoing coronary angiography (CAG) (Mai et al.), prevalence of malnutrition was also high and malnutrition-associated risk of mortality was higher in patients with left ventricular ejection fraction (LVEF) ≥40% than those with <40%, highlighting the need of giving greater attention to malnutrition in these patients. The impact of nutritional risk not only on health outcomes but hospital costs were also addressed by Liu et al. in a National Study of an Asian population.

Considering the potential utility of hypoalbuminemia as a therapeutic target for atrial fibrillation risk reduction, Wang et al. conducted a meta-analysis to assess the relationship between albumin and atrial fibrillation and the potential dose-response effect, demonstrating that a low serum albumin level was significantly associated with an increased risk of AF, paving the way for further studies aimed at exploring the effects of interventions to increase serum albumin levels for the prevention of AF. Nasab et al. also focused on the intervention aspects of malnutrition, particularly the differences between the feeding indications provided by a dietitian and a surgeon in patients undergoing gastrointestinal surgery who received parenteral nutrition. Results pointed out the relevance of incorporating a dietitian in the care of patients being arterially fed.

Finally, a valuable contribution to the field was provided by Marunowski et al., who proposed normative reference values for subcutaneous and visceral fat based on magnetic resonance imaging in a pediatric population, these are relevant data for the assessment of nutritional status in this Caucasian population from Poland.

Considering all mentioned contributions, it is evident that malnutrition is a topic of paramount interest in the health field. In the context of chronic disease, sarcopenia, muscle function (handgrip strength), muscle composition (low skeletal muscle mass index and low skeletal muscle radiodensity), and phase angle are all parameters that may be useful for nutritional assessment and monitoring since they are associated with clinically relevant outcomes and overall survival. Further research could be oriented toward developing population-specific normative values for muscle function and composition for a more accurate nutritional assessment in diverse patient populations.

## Author Contributions

EC-R wrote the introduction and central part with comments to the cited papers and references. LC-M wrote the conclusion and reviewed/edited the introduction and central part. All authors contributed to the article and approved the submitted version.

## Conflict of Interest

The authors declare that the research was conducted in the absence of any commercial or financial relationships that could be construed as a potential conflict of interest.

## Publisher's Note

All claims expressed in this article are solely those of the authors and do not necessarily represent those of their affiliated organizations, or those of the publisher, the editors and the reviewers. Any product that may be evaluated in this article, or claim that may be made by its manufacturer, is not guaranteed or endorsed by the publisher.
